# What goes on in the resting-state? A qualitative glimpse into resting-state experience in the scanner

**DOI:** 10.3389/fpsyg.2015.01535

**Published:** 2015-10-08

**Authors:** Russell T. Hurlburt, Ben Alderson-Day, Charles Fernyhough, Simone Kühn

**Affiliations:** ^1^Department of Psychology, University of Nevada, Las VegasLas Vegas, NV, USA; ^2^Department of Psychology, Durham UniversityDurham, UK; ^3^Center for Lifespan Psychology, Max Planck Institute for Human DevelopmentBerlin, Germany; ^4^Clinic and Polyclinic for Psychiatry and Psychotherapy, University Clinic Hamburg-EppendorfHamburg, Germany

**Keywords:** resting state, descriptive experience sampling (DES), fMRI, default mode network (DMN), Resting State Questionnaire (ReSQ), mind wandering

## Abstract

The brain’s resting-state has attracted considerable interest in recent years, but currently little is known either about typical experience during the resting-state or about whether there are inter-individual differences in resting-state phenomenology. We used descriptive experience sampling (DES) in an attempt to apprehend high fidelity glimpses of the inner experience of five participants in an extended fMRI study. Results showed that the inner experiences and the neural activation patterns (as quantified by amplitude of low frequency fluctuations analysis) of the five participants were largely consistent across time, suggesting that our extended-duration scanner sessions were broadly similar to typical resting-state sessions. However, there were very large individual differences in inner phenomena, suggesting that the resting-state itself may differ substantially from one participant to the next. We describe these individual differences in experiential characteristics and display some typical moments of resting-state experience. We also show that retrospective characterizations of phenomena can often be very different from moment-by-moment reports. We discuss implications for the assessment of inner experience in neuroimaging studies more generally, concluding that it may be possible to use fMRI to investigate neural correlates of phenomena apprehended in high fidelity.

## Introduction

In resting-state functional magnetic resonance imaging (rfMRI), spontaneous changes in the blood oxygen dependent (BOLD) signal can be used to study networks of brain areas that are functionally connected and tend to co-activate when a participant is not performing any explicit task, that is, when a participant is in what is often referred to as the “resting state.” These studies produce activity in a consistent network of brain regions, including lower precuneus, superior and inferior anterior medial frontal regions, and posterior lateral parietal cortices (Gusnard and Raichle, 2001). Initially, these regions were identified because they were found to be consistently deactivated when tasks are performed. The consistency with which this set of brain regions decrease in activity during tasks and increase during fixation or resting has led to the notion of a so-called “default mode” network of the brain (Buckner et al., 2008).

Scientists interested in rfMRI have provided a wide variety of characterizations of the kinds of experiences and processes that are ongoing when default-mode brain regions are active, as exemplified in **Table 1**. As those characterizations demonstrate, there is wide variability in the descriptions of phenomena in the resting state. Researchers are increasingly sensitive to the potential phenomenological heterogeneity of subjective experience in the resting state (Smallwood and Schooler, 2015), denoted here as ‘mind wandering’ in accord with popular usage (Callard et al., 2013). For example, Gorgolewski et al. (2014) demonstrated relations between the content and form of self-generated thoughts in the resting state (such as imagery and future-related thinking) and specific intrinsic neural activity patterns. Ruby et al. (2013) showed that the relation between the emotional content of thoughts and subsequent mood was modulated by the socio-temporal content of the thoughts, specifically their relatedness to the past. At the same time, there is a growing recognition that psychological experiences collected under the umbrella of mind wandering are not necessarily the experiential manifestation of neural activity in the resting state, and that the relation between experiential phenomena and neural state is complex (Raichle, 2011; Fox and Christoff, 2014).

**Table 1:** Characterizations of experiences when the default mode is active.

	Characterization	Source
1	“unconstrained verbally mediated thoughts”	Shulman et al., 1997, p. 648
2	“semantic knowledge retrieval, representation in awareness, and directed manipulation of represented knowledge for organization, problem-solving, and planning”	Binder et al., 1999, p. 80
3	“active retrieval of past experiences and planning of future experiences”	Andreasen et al., 1995, p. 1576
4	“retrieval and manipulation of past events, both personal and general, in an effort to solve problems and develop future plans”	Greicius et al., 2003, p. 257
5	“enhanced watchfulness toward the external environment (e.g., waiting for upcoming task-relevant stimuli or attending to scanner noise and incidental light)”	Gilbert et al., 2007, p. 43
6	“inner thought, self-reflective thinking in terms of planning for the future, or simulation of behavior… interrupted… into a… extrospective… state of mind… characterized… as increased attention and readiness,… sensorimotor planning for future routes of action in response to potential changes in the inner and outer environment”	Fransson, 2005, p. 26
7	“not focused on the external environment,… internally focused tasks including autobiographical memory retrieval, envisioning the future, and conceiving the perspectives of others”	Buckner et al., 2008, p. 1
8	“spontaneous, internally directed cognitive processes”	Andrews-Hanna et al., 2010, p. 322
9	“spontaneous mental contents which are unrelated to perception and coordinate[d]… so that they are maintained in the face of competing sensory information”	Smallwood et al., 2012, p. 67
10	“an ultimate state of inspection of the self”	Wicker et al., 2003, p. 229
11	“stable, unified perspective of the organism relative to its environment (a ‘self’)”	Gusnard and Raichle, 2001, p. 692

Most characterizations of mind wandering as the psychological counterpart of the brain’s resting state follow from indirect theoretical considerations (researchers set tasks for participants in the scanner and then theorize about what is *not* ongoing in those tasks; Callard and Margulies, 2014) or retrospective characterizations. Researchers have recently developed three questionnaires that ask participants retrospectively to characterize their resting state cognition: the Resting State Questionnaire (ReSQ; Delamillieure et al., 2010), the Amsterdam Resting State Questionnaire (ARSQ; Diaz et al., 2013, and its revised and extended counterpart the ARSQ 2.0 Diaz et al., 2014), and the New York Cognition Questionnaire (NYC-Q; Gorgolewski et al., 2014). These questionnaires typically ask volunteers to participate in an fMRI resting-state session (or online analog thereof; Diaz et al., 2014), exit the scanner, and then immediately characterize their in-scanner resting state experience. The ReSQ asks participants to use visual-analog scales to estimate the proportion of their resting-state time that they had been engaged in visual imagery, in inner language, in somatosensory awareness, in inner musical experience, and in the mental manipulation of numbers. The ARSQ 2.0 uses 30 Likert-scale items divided into ten factors (discontinuity of mind, theory of mind, self, planning, sleepiness, comfort, somatic awareness, health concern, visual thought, and verbal thought). The NYC-Q asks participants to report on the content and form of their self-generated thoughts using Likert scales. Factor analysis of the NYC-Q has revealed five main content factors of resting-state experiences: past, future, positive, negative, and social experiences, and three main form factors: words, images, and thought specificity. All three of these questionnaires ask respondents to characterize their resting state in general and therefore do not characterize particular moments. However, Hurlburt and Heavey (2015) argued that retrospective questionnaires may not be adequate to characterize moments of inner experience. They held that retrospective reports about inner experience are perhaps more influenced by the participant’s presuppositions about experience than by the participant’s experience itself, that retrospections are skewed by reporting biases such as recency or salience, and so on.

There have been a few studies that have sought to overcome the retrospectiveness limitations by using experience sampling to examine experiences at specific moments during the resting state. For example, Christoff et al. (2009) had subjects in an fMRI scanner perform a boring go/no-go task and intermittently presented thought probes. Each probe asked participants to report on their mental state using two Likert scales: one asked whether attention was focused on the task (rated from *completely on task* to *completely off task*); the second asked whether the subject was aware of where their attention was focused (rated from *completely aware* to *completely unaware*). However, such studies have focused on rating one or two aspects of experience and have not tried to provide descriptions of actual ongoing experience.

Thus there are to date no investigations that have sought to provide high fidelity descriptions of the phenomena that are ongoing in the resting state. Understanding the experiential details of the resting state is important because the resting state is typically the baseline against which the results of particular tasks are compared, as well as being an important target of investigation in its own right. Hurlburt and Heavey (2015) suggested that descriptive experience sampling (DES; Hurlburt, 1990, 1993, 2011a; Hurlburt and Akhter, 2006; Hurlburt and Heavey, 2006; Hurlburt and Schwitzgebel, 2007) may be capable of producing high fidelity descriptions of experience. In its typical application, DES uses a random beeper to interrupt participants in their natural environments. Participants are to attend to the experience that was ongoing at the moment of the onset of the beep and to jot down notes about that experience. A typical participant receives six such beeps in typically a 3-h window. Later that day (or the next day), the participant meets with the investigator in what DES calls an expositional interview designed to discover the details of the six experiences and “iteratively” to improve the quality of subsequent sampling. The sample/interview procedure is then repeated over a number (typically 4–6) of sampling days.

Descriptive experience sampling has been compared to and contrasted with a variety of qualitative and related methods (see **Table 2**). In broad strokes, DES differs from other methods in that DES aims at pristine inner experience (Hurlburt, 2011a), experience that is directly apprehendable at a moment; its view that people often do *not* know the characteristics of their own experience unless trained to apprehend them, probably requiring an iterative method (Hurlburt, 2009, 2011a); its minimization of retrospection; and its methods of bracketing of presuppositions (Hurlburt and Schwitzgebel, 2007, 2011; Hurlburt, 2011a).

**Table 2:** Comparing DES with qualitative and related methods.

Method	Comparison reference
Kvales’ qualitative research interview	Hurlburt and Heavey, 2006, Chap. 12
Giorgi’s phenomenological psychology	Hurlburt and Heavey, 2006, Chap. 12
Stern’s micro-analytic interview	Hurlburt, 2011a, Chap. 7
Vermersch’s explicitation interview	Hurlburt, 2011b
Petitmengin’s second-person interview	Hurlburt and Akhter, 2006
van Manen’s hermeneutic phenomenological inquiry	Heavey et al., 2010
Moustakas’s human science research	Heavey et al., 2010
Armchair introspection	Hurlburt and Schwitzgebel, 2007, Chap. 11; 2011
Eyewitness testimony	Hurlburt and Schwitzgebel, 2007, Chap. 11
Questionnaires	Hurlburt and Heavey, 2015
Non-DES experience sampling methods	Hurlburt and Heavey, 2015

**FIGURE 1:**
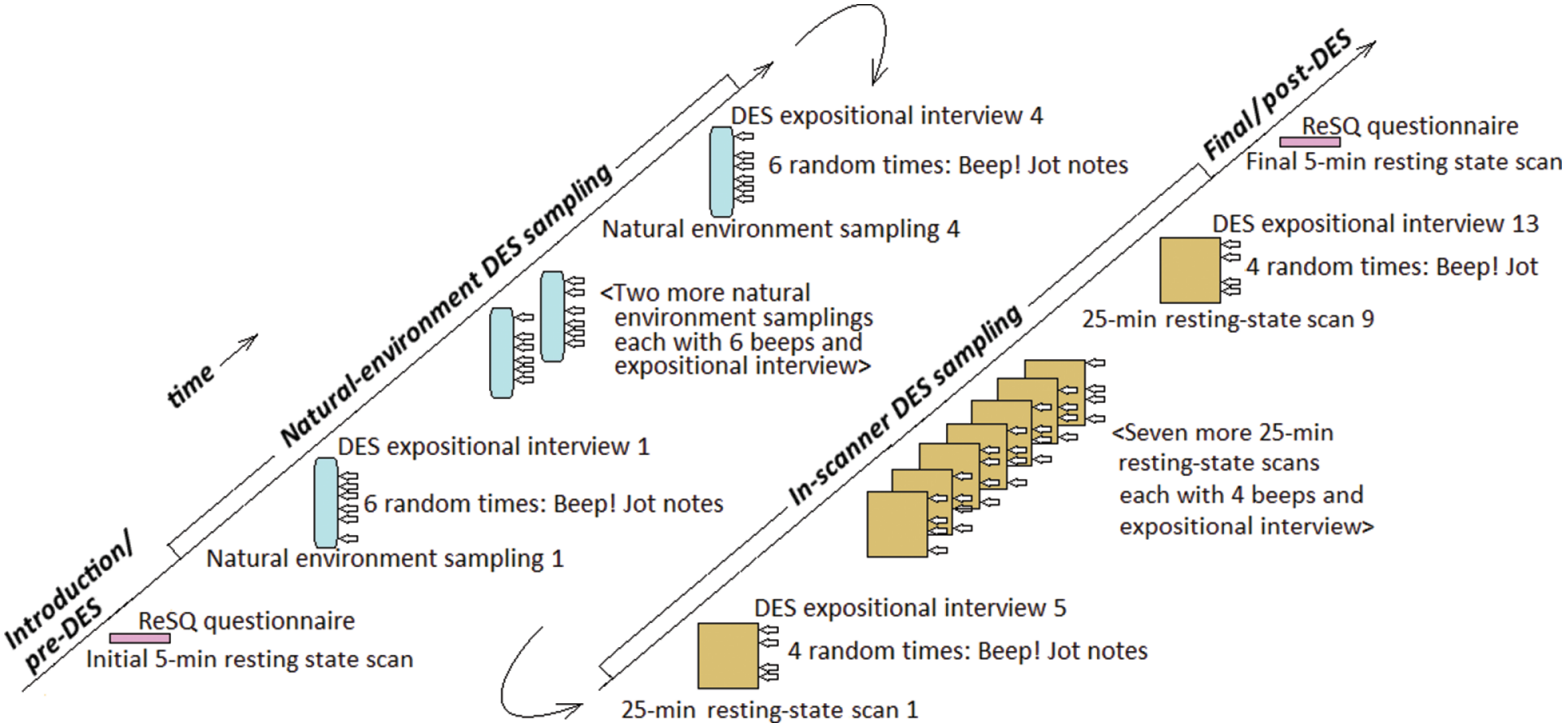
**Procedure schematic for each participant**.

We have seen, then, that (a) it would be desirable to understand the phenomenology of experience while in the resting state in the scanner; and that (b) DES, with its iterative training, may provide high fidelity access to the phenomenology of experience in a way that can be integrated with fMRI (Kühn et al., 2014). The present study seeks to combine those two considerations, a non-trivial exercise because the duration of DES studies (typically measured in days) is far greater than is typically available in the magnetic resonance imaging (MRI) resting state sessions (typically measured in minutes). As illustrated in **Figure 1**, the present study attempted to overcome that obstacle first by preliminarily training participants in DES in their natural environments prior to involvement in the scanner and then by using DES in extended-duration fMRI resting state sessions. The extended-duration resting-state sessions involved nine 25-min fMRI sessions for each participant (9 × 25 = 225 min of resting state per participant instead of the more usual 5 or 10 min). Participants were given the same instructions as are usual in resting-state studies.

## Materials and Methods

### Participants

Five native English-speaking (because RTH would be the lead interviewer) participants who currently lived in Berlin (because MRI scanning would take place at the Max Planck Institut für Bildungsforschung) participated on the basis of informed consent and with ethical committee approval according to the Declaration of Helsinki. All participants had normal or corrected-to-normal vision. No participant had a history of neurological, major medical, or psychiatric disorder. The participants (three females, two males) had a mean age of 22.4 (ranging from 18 to 30) and all but one (male) were right-handed.

### Measures

The ReSQ (Delamillieure et al., 2010) is a semi-structured questionnaire that asks participants to characterize their inner experiences when they had been resting quietly in a MRI scanner.

Descriptive experience sampling was performed as described in Hurlburt (2011a) and elsewhere. DES is primarily an idiographic procedure, allowing and encouraging the examination of phenomena that may be idiosyncratic to particular individuals, but it has identified five phenomena that are characteristic of many individuals, which we will call the 5FP (five frequent phenomena): inner speaking (the experience of speaking to oneself in one’s own voice but without any external sound or motor movement; Hurlburt et al., 2013); inner seeing (experiencing imaginary seeing); unsymbolized thinking (the directly apprehended experience of thinking that is not accompanied by the apprehension of words, visual images, or any other symbols; Hurlburt and Akhter, 2008a,b); sensory awareness (experience where a particular focus is on a sensation, not for any instrumental utility; Hurlburt et al., 2009); and feelings (the experience of emotion; Heavey et al., 2012).

Other measures not relevant here were administered as part of a larger study.

### Procedure

A bird’s-eye view of the procedure for each participant is illustrated in **Figure 1**. Each participant was scheduled for 19 sessions, generally across a 2-weeks period, which were divided into four phases. Schedules were individualized for each participant; for example, scanner sessions were generally twice a day, but because of holiday or other pressures were occasionally once or three times on some days. DES instructions were also individualized; a tenet of DES is to be candid, and participants with more questions got more initial instructions.

In the *introduction/pre-DES resting state* phase (**Figure 1**, bottom left), we fully explained the study, administered initial questionnaires not relevant to the present report, and familiarized the participant with the MRI scanner and procedures. Then the participant entered the scanner, where we conducted a structural scan and then a 5-min resting state scan according to standard procedures. The resting-state instructions were “please close your eyes and relax, without falling asleep.” Immediately following the resting-state scan, the participant exited the scanner and filled out the ReSQ questionnaire under supervision of a psychologist.

In the *natural-environment DES sampling* phase (**Figure 1**, middle left), which began typically immediately after the completion of the ReSQ, we instructed the participant in the use of the DES beeper and the sampling task (Hurlburt, 2011a; Hurlburt et al., 2013): the participant was to wear the beeper in the participant’s natural environment for ∼3 h, during which the participant would hear (through an earphone) six randomly occurring beeps. Immediately after each beep the participant was to jot down notes (in a supplied small notebook) about the ongoing inner experience—the experience that was “in flight” at the moment the beep sounded. Following the DES instructions, the participant proceeded to the natural environment, wore the DES beeper, and, when beeped, collected six experience samples. Later that day or the next day the participant returned for the first DES expositional interview about those six beeped experiences; this interview was conducted by RTH and at least one and as many as four additional interviewers (the study was part of a training program), usually including SK and sometimes CF or BA-D. The expositional interview (following the DES procedure) was “iterative” (Hurlburt, 2009, 2011a), designed to increase, across sampling days, the participant’s skill in apprehending and describing inner experience. Following this interview, the participant returned to his or her everyday environment and responded to six more random beeps, again jotting notes about the ongoing experiences. The following day the participant returned for a second expositional interview about the second-sampling-day’s six beeped experiences. This sequence was repeated twice more, so that the participant sampled in a total of four natural-environment periods, each followed by an (iterative) expositional interview. Theoretically, this procedure could produce 4 × 6 = 24 natural-environment experience samples. We (as is typical in DES) considered the first day’s samples unreliable and discarded them from subsequent analysis. The remaining days often produce less than the maximum six samples because the 1-h expositional interview runs out of time before all samples are discussed. Undiscussed samples are discarded. Hurlburt (2011a) has found that four such iterative sampling day/expositional interviews typically result in skill acquisition adequate for the remainder of this study; however, more interviews could be scheduled if the expositional interviews suggested it; one participant (#3) had one additional sampling day to ensure we understood each other about what was or was not inner speaking. This procedure resulted in a variable number of natural-environment samples, ranging from 13 to 22.

In the *in-scanner DES sampling* phase (**Figure 1**, middle right), the participant (having been trained in DES in the natural environment) entered the scanner for a 25-min session with resting-state instructions “please relax, without falling asleep and do keep your eyes open.” At four quasi-random times, the participant received a DES beep through a headphone (just as in the natural environment except the in-scanner beep automatically terminated after 1.5 s, whereas the natural environment beeps must be terminated by the participant). Immediately after each beep, the participant jotted a few notes about the ongoing experience on a clipboard positioned on the lap (viewable through a mirror). Immediately after exiting the scanner, the participant participated in a DES expositional interview about the four randomly beeped experiences. This in-scanner sequence (25-min fMRI scan/four beeps with jotted notes/expositional interview) was repeated eight more times, typically spread over five days, resulting in 9 × 4 = 36 random samples of experience occurring in 9 × 25 = 225 min of fMRI scanning for each participant.

In the *final/post DES* phase (**Figure 1**, upper right), the participant entered the scanner for another structural scan and a final 5-min standard resting-state scan using the same instructions and procedures as in the first 5-min resting state scan. The participant then filled out the ReSQ questionnaire under supervision of a psychologist and then was candidly debriefed.

#### DES Quantitative Analysis

RTH and at least one additional person present at the interview independently judged whether each of the 5FP was present at each sample; discrepancies were resolved by discussion.

#### Scanning Procedure

Images were collected on a 3T Magnetom Trio MRI scanner system (Siemens Medical Systems, Erlangen, Germany) using a 32-channel radio frequency head coil. Structural images were obtained using a three-dimensional T1-weighted magnetization-prepared gradient-echo sequence (MPRAGE) based on the ADNI protocol^1^ (repetition time [TR] = 2500 ms; echo time [TE] = 4.77 ms; TI = 1100 ms, acquisition matrix = 256 × 256 × 176, flip angle = 7°; 1 mm × 1 mm × 1 mm voxel size). Functional images were collected using a T2*-weighted echo planar imaging (EPI) sequence sensitive to blood oxygen level dependent (BOLD) contrast (TR = 2000 ms, TE = 30 ms, image matrix = 64 × 64, FOV = 216 mm, flip angle = 80°, voxel size 3 mm × 3 mm × 3 mm, 36 axial slices).

#### Resting State fMRI Analysis

The first 10 volumes were discarded to allow the magnetisation to approach a dynamic equilibrium and for the participants to get used to the scanner noise. Part of the data pre-processing, including slice timing, head motion correction (a least squares approach and a six-parameter spatial transformation) and spatial normalization to the Montreal Neurological Institute (MNI) template (resampling voxel size of 3 mm × 3 mm × 3 mm), were conducted using the SPM5 and Data Processing Assistant for Resting-State fMRI (DPARSF, Chao-Gan and Yu-Feng, 2010). A spatial filter of 4 mm FWHM (full-width at half maximum) was used. Participants showing head motion above 3.0 mm of maximal translation (in any direction of *x*, *y*, or *z*) or 1.0° of maximal rotation throughout the course of scanning would have been excluded; this was not necessary.

After pre-processing, linear trends were removed. Then the fMRI data were temporally band-pass filtered (0.01–0.08 Hz) to reduce the very low-frequency drift and high-frequency respiratory and cardiac noise (Biswal et al., 1995). Amplitude of low frequency fluctuations (ALFFs) analysis (Yang et al., 2007; Zang et al., 2007) was performed using DPARSF (Chao-Gan and Yu-Feng, 2010). We chose ALFF analysis since it is a commonly used metric with high test-retest reliability (Zuo and Xing, 2014). The time series for each voxel was transformed to the frequency domain using fast Fourier transform (FFT), and the power spectrum was obtained. Because the power of a given frequency is proportional to the square of the amplitude of this frequency component in the original time series in the time domain, the power spectrum obtained by FFT was square rooted and then averaged across 0.01–0.08 Hz at each voxel. This averaged square root is the ALFF (Zang et al., 2007). The ALFF of each voxel was divided by the individual global mean of ALFF within a brain-mask, which was obtained by removing the tissues outside the brain using software MRIcro (by Chris Rorden^2^). A height threshold of *p* < 0.001 was applied to the *t* maps.

We performed the same ALFF analysis on the fMRI data acquired during the extended-duration resting-state sessions except that we removed the images between the onset of the DES beep and 2 min thereafter to exclude activity elicited by tone presentation and the subsequent motor activation while the subjects were jotting down notes. However, we also conducted the ALFF analysis on all extended-duration resting state data, including the 2 min after each beep.

## Results

### Characterization of Participants

**Table 3** shows the percentages of each of the five frequent phenomena (5FP) that had been described by Heavey and Hurlburt (2008). These percentages are shown for each participant, divided into pairs of columns for the natural environment sampling and the in-scanner resting state sampling. We begin by considering the natural environment.

**Table 3:** Natural environment and in-scanner resting-state inner experience characteristics (5FP^a^ percentages^b^) for each participant.

	Inner speaking		Inner seeing		Unsymbolized thinking		Sensory awareness		Feelings	
					
Participant	Nat. env.	Scanner rest. st.	Nat. env.	Scanner rest. st.	Nat. env.	Scanner rest. st.	Nat. env.	Scanner rest. st.	Nat. env.	Scanner rest. st.
1 (*n* = 13^c^)	8	17	85	19	0	8	85	75	23	3
2 (*n* = 16)	13	22	0	22	6	3	94	75	13	3
3 (*n* = 22)	23	53	0	25	5	17	68	47	14	3
4 (*n* = 18)	39	39	6	42	11	33	6	19	72	22
5 (*n* = 12)	8	14	33	67	25	39	75	78	25	11
**Mean**	18.2	29.0	24.8	35.0	9.4	20.0	65.6	58.8	29.4	8.4
χ^2^ (*df* = 4)	6.99	19.09	47.54	24.57	6.02	22.01	35.56	38.18	21.00	13.82
*p* (for χ^2^)	0.13	0.001	0.0001	0.0001	0.20	0.001	0.000001	0.000001	0.001	0.01
					
*r* (*df* = 3)	0.73^d^		-0.09		0.84		0.93		0.95	

*^a^5FP = “five frequent phenomena” as discussed by Heavey and Hurlburt (2008). ^b^Percentages do not add to 100 because experiences can have more than one characteristic. ^c^Number of natural environment samples. Each participant had 36 in-scanner samples. ^d^Pearson r between Nat. env. and Scanner rest. st. across participants.*

#### Characteristics of Our Participants in their Natural Environments

The study design called for each participant to undergo 4 days [or more if the interviews called for it; one participant (#3) had five natural environment days] of DES sampling in their natural environments, for a potential maximum of 18 samples (24 for participant #3) after discarding the first day. The first column of **Table 3** shows (in parenthesis) the number of natural environment samples we actually obtained for each participant after discarding the first day. The number of natural environment samples varied from participant to participant because training is individualized.

The remaining columns of **Table 3** show the percentages of each of the 5FP for each participant. For example, in the natural environment, participant #1 had 1 instance of inner speaking out of his 13 natural environment samples, so the upper left cell shows 100(1/13) = 8%.

The **Table 3** row labeled “Mean” shows that, on average, sensory awareness was our participants’ most frequently occurring phenomenon in the natural environment (65.6%; we consider in-scanner percentages below). Our participants thus had more frequent sensory awareness than might be expected from Heavey and Hurlburt’s (2008) stratified natural environment sample, where only 22% of samples involved sensory awareness. Inner speaking (18.2%), inner seeing (24.8%), and feelings (29.4%) each occurred in the natural environment at about the same frequency found by Heavey and Hurlburt (26, 34, and 26%, respectively). Unsymbolized thinking occurred at a somewhat lower frequency in the natural environment than might be expected from Heavey and Hurlburt (9.4% instead of Heavey and Hurlburt’s 22%).

#### Our Participants Differ from each other in their Natural Environments

Now we ask whether our participants differ from each other on the 5FP (Heavey and Hurlburt, 2008, had reported large individual differences in 5FP characteristics). This is an exploratory study, so we conducted a separate examination of each of the 5FP characteristics. We discovered, for example, that our five participants had very different percentages of sensory awareness when sampled in the natural environment (χ^2^ = 35.56, *p* = 0.000001, *df* = 4). Similar analyses for the other four 5FP phenomena revealed substantial individual differences for inner seeing and for feelings; the inner speaking and unsymbolized thinking differences were smaller. Some individual differences among our participants are large: for example, the natural-environment frequency of sensory awareness ranged from 6% (for participant #4) to 94% (for participant #2); the frequency of inner seeing ranged from 0% (for participants #2 and #3) to 85% (for participant #1), and so on. We performed subsequent analyses in a variety of ways, always with the same results. For example, participant 1 is left handed; if we exclude him from the data, we similarly conclude that the remaining four right-handed people are different from each other in the same ways as all five are different.

#### Our Participants Differ from each other in the In-Scanner Resting State Sessions

We now consider the in-scanner resting state percentages shown in **Table 3**, asking whether our participants differed from each other in the scanner resting state. We performed the same chi-squared analyses as just discussed, finding that participants in the scanner differed greatly from one another on the frequencies of all five of the 5FP phenomena. We conclude that our participants’ inner experiences are different from each other in the resting state. These differences are large: for example, the resting state frequency of sensory awareness ranged from 19% (for participant #4) to 78% (for participant #5); the frequency of inner seeing ranged from 19% (for participant #1) to 67% (for participant #5), and so on. We again performed subsequent analyses in a variety of ways, always with the same results. These results were similar to and more striking than the natural environment differences (probably because the natural environments themselves differed greatly from one participant to the next, and because the iterative nature of DES makes early sampling days (which, in this study, were all natural environment samplings) results relatively unreliable compared to later sampling days (which were all in the scanner).

#### Our Participants’ Experiences in the Resting State Sessions are Broadly Similar to their Experiences in the Natural Environment

We now turn to consider whether a participant’s in-scanner resting state percentages shown in **Table 3** are similar to that participant’s percentages in their natural environments. We display an exploratory/descriptive Pearson correlation for each of the 5FP across participants (*df* = 3) in the last row of **Table 3**; these correlations are all very high except for inner seeing, which is approximately zero. The high correlations between in-scanner resting state and natural environment does not imply that experience frequency is the same in the scanner and the natural environment. It implies that participants who had a relatively high frequency of a particular characteristic in the natural environment also have a relatively high frequency in the scanner. For example, there were, overall, fewer feelings in the scanner (8.4%) than in the natural environment (29.4%), but the participants who had the most feelings in the natural environment (#4 and 5) also had the most feelings in the scanner. The same, but opposite direction, is true for unsymbolized thinking: there was more unsymbolized thinking in the scanner (20.0%) than in the natural environment (9.4%), but the participants who had the most unsymbolized thinking in the natural environment (#4 and 5) also had the most unsymbolized thinking in the scanner.

The distributions of experiential percentages are sometimes very skewed, which may inflate a Pearson correlation, so we conducted the same analyses using Spearman rank correlations, which are not affected by skew. The Spearman correlations (0.87, -0.05, 0.70, 0.67, and 0.89, respectively) are very similar to the Pearson correlations shown in the bottom row of **Table 3**, suggesting that the correlations between natural environment and resting state phenomena were not due to the extremity of the values.

Thus our data suggest that for these participants, inner experience in the resting state is quite strongly related to inner experience in the natural environment except for the case of inner seeing. We re-emphasize the exploratory nature of these correlations given the small sample size.

### Are Extended-Duration Resting State Sessions Experientially Similar to Typical Resting State Sessions?

Next we ask whether our extended-duration resting state sessions are, broadly speaking, similar to typical (5- or 10-min) resting-state sessions. Our resting state sessions were extended in two ways: there were multiple sessions (nine) rather than the more typical one, and each session had a long duration (25 min) rather than the more typical 5 or 10 min; therefore we must explore the potential impact of each of those two kinds of extended duration.

#### There was Little Drift Across the Multiple Resting State Sessions

First, we investigate whether the 5FP experiential frequencies altered or drifted across this study’s multiple (nine) resting state sessions. **Table 4** shows the percentage of samples in each 5FP category, aggregated across participants, displayed by session number. There were four samples for each of the five participants in each session, thus 4 × 5 = 20 samples per session. Across all participants in the first session, there were, for example, four instances of inner speaking, so 4/20 = 20% is displayed in the upper left (session 1/inner speaking) cell of **Table 4**. As shown in the middle panel of **Table 4**, a chi-squared test of proportions suggests that our participants’ frequency of inner speaking was independent of session (χ^2^ = 7.68, *p* = 0.47, *df* = 8). The results are similar for the remaining four 5FP characteristics, as shown in the “All sessions χ^2^(*df* = 8)” rows of **Table 4**. These χ^2^ statistics are all very small (and the corresponding *p* values large), far from indicating any systematic differences, except for feelings, where χ^2^ = 20.95 (*p* = 0.01).

**Table 4:** Inner experience characteristics (5FP percentages^a^) aggregated across participants by scanner resting-state session.

Session	Inner speaking	Inner seeing	Unsymbolized thinking	Sensory awareness	Feelings
1	20	25	40	50	5
2	30	20	15	40	5
3	45	40	15	70	0
4	40	45	5	75	15
5	15	20	25	70	0
6	25	40	20	65	15
7	35	45	20	60	0
8	30	35	25	60	30
9	20	45	15	40	5
**Mean**	28.89	35.00	20.00	58.89	8.33
All sessions					
χ^2^ (*df* = 8)	7.68	7.91	9.38	11.06	20.95
*p* (for χ^2^)	0.47	0.44	0.31	0.20	0.01
*r* (*df* = 7)	-0.16	0.56	-0.21	-0.03	0.34
1st session					
χ^2^ (*df* = 1)	0.87	0.99	5.63	0.73	0.33
*p* (for χ^2^)	0.35	0.32	0.02	0.39	0.57

*^a^Percentages do not add to 100 because experiences can have more than one characteristic.*

We also asked whether there was a consistent trend across sessions by computing an exploratory/descriptive Pearson correlation between the percentage and the session number; the “*r* (*df* = 7)” row of **Table 4** shows that those correlations are quite small. The strongest correlation (0.56) was for inner seeing, indicating that inner seeing increased in frequency across scanner sessions.

The first of the nine resting state sessions is arguably the session most similar to a typical one-shot resting state study. As shown in the bottom panel of **Table 4**, we asked whether the 5FP characteristics in the first 25-min resting state session differed from the 5FP characteristics in the remaining eight 25-min resting state sessions. There were no large differences, although there was somewhat more unsymbolized thinking in the first session.

We performed these χ^2^ analyses in several different ways, all discovering approximately the same level of independence. For example, we also asked whether the last session differed from the first eight; and whether the first four sessions differed from the last four. None of these tests (not reported here) showed large differences.

Taken together, these results indicate that there were not large or systematic shifts in experience across the nine resting state scanner sessions.

#### There was Little Drift within the Long Duration Resting State Sessions

Second, we investigate whether our participants’ 5FP experiential frequencies altered or drifted within each of this study’s long duration (25-min) resting state sessions. **Table 5** shows the aggregation of samples from the first half of each session and those from the second half. For example, we asked whether those aggregates differed for inner speaking and found that there was somewhat more inner speaking in the first half of sessions (37% vs. 21%; χ^2^ = 5.3, *p* = 0.02, *df* = 1). The other 5FP characteristics had no large differences, as shown in the bottom two rows of **Table 5**.

**Table 5:** Inner experience characteristics (5FP percentages^a^) in first half or second half of scanner sessions.

Session part	Inner speaking	Inner seeing	Unsymbolized thinking	Sensory awareness	Feelings
First half	37	31	18	60	6
Second half	21	39	22	58	11
χ^2^ (*df* = 1)	5.30	1.20	0.56	0.09	1.82
*p*	0.02	0.27	0.46	0.76	0.18

*^a^Percentages do not add to 100 because experiences can have more than one characteristic.*

Furthermore, we performed all the analyses described for **Tables 2** and **3** within each participant singly, finding similar levels of independence within participants. The *p*-values for individual participants when performing the kind of analysis shown in **Table 4** are 0.71, 0.56, 0.47, 0.21, and 0.83. Similarly, the individual participant *p* values comparable to the **Table 5** analysis were 0.68, 0.54, 0.77, 0.02, and 0.98. There is one small *p* value in those studies: participant #4 can be characterized as having less inner speaking and more inner seeing aggregated across the second halves of all sessions. That is, however, the only relatively large frequency difference.

Thus our data suggest that there are not large or systematic shifts in experience within the nine resting state scanner sessions.

### Are Extended-Duration Resting State Sessions Neurophysiologically Similar to Typical Resting State Sessions?

**FIGURE 2:**
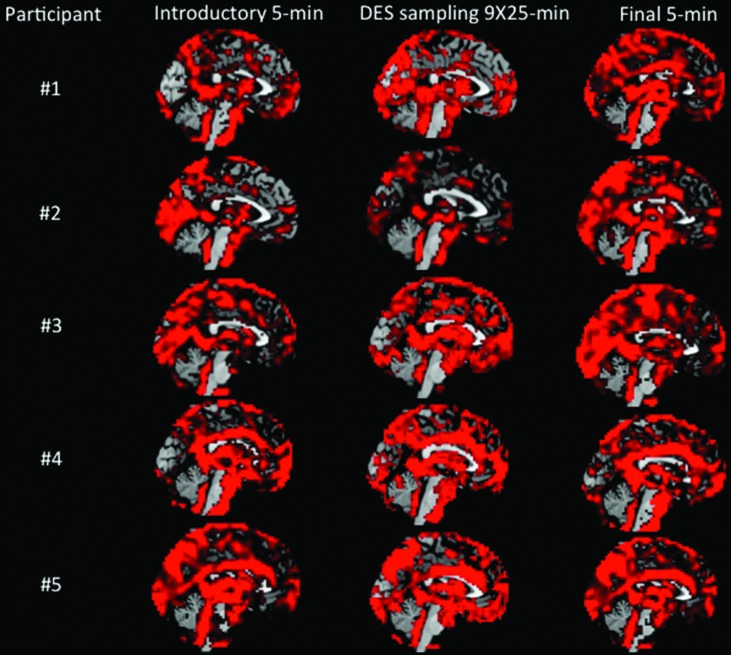
**Amplitude of low frequency fluctuation (ALFF) maps for each participant in the three conditions**.

We have explored whether there were *experiential* characteristics that would lead us to suspect that our extended-duration resting state sessions were substantially different from typical resting state sessions, and concluded that there were not. Now we ask whether there were *neurophysiological* characteristics that would suggest a difference between extended-duration resting state sessions and typical resting state sessions. The small sample size makes customary statistical analysis of fMRI data unsatisfactory, but we can examine in broad strokes whether the brain activity we recorded is similar to the default mode brain activity generally understood to be ongoing in typical resting state scanner studies. As illustrated above in **Figure 1**, our design had a standard 5-min resting state scan in the *introduction/pre-DES resting state* phase and another standard 5-min resting state scan in the *final/post-DES* phase. Our resting state sessions in the *in-scanner DES sampling* phase used typical resting state instructions except for the times participants spent jotting down notes in response to the DES random beeps. As shown in **Figure 2**, we computed ALFF from the data of each participant for each of the three phases, arbitrarily excluding 2 min beginning with the onset of each beep in the *in-scanner DES sampling* phase to exclude brain activity related to the beep and subsequent motor activity. In all 15 plots we find higher ALFF values in the regions typically activated in resting state studies, namely in superior and inferior anterior medial frontal regions, lower precuneus, and posterior lateral parietal cortex. On the possibility that excising the 2 min following each beep creates an artifact, we computed the same 15 plots including the 2 min after each beep in the analysis; those plots are nearly identical to those displayed in **Figure 2** (and are available from the authors).

### Phenomena in the Resting State

We inquire now about the phenomena that DES apprehended during the extended-duration resting state scanner sessions. We begin by recalling one of the main findings of **Table 3** above: there are great individual differences in people’s resting state experience. The resting state frequency of inner speaking ranged from 17 to 53%; inner seeing ranged from 19 to 67%; sensory awareness ranged from 19 to 78%. Thus our data suggest that there is *no* single kind of resting state phenomenology.

To give a glimpse into the kinds of experience that are found in the resting state, we provide a few typical examples, one from each participant. The entire set of experiences is available from the authors.

(1) (Jack, participant 1, sample 8.3) Jack is rubbing with his thumb the fabric that holds the writing board, and he feels the snagging of the fabric on his thumb. This is more a sense in his thumb than of the fabric. He is involved with determining whether this is wool or synthetic, but that is a part of the sensation, not a cognitive act. (Sensory awareness).
(2) (Lara, participant 2, sample 7.7) Lara is looking at the edges of the scanner mirror—left bottom corner, and sees two of them, layering. Simultaneously she is hearing herself say, to no one in particular, “I really want to talk to you.” The voice is recognized to be her own, expressed in her own natural way; however, the vocal characteristics are not of her own voice but of some female voice that she doesn’t recognize. The wrongness of the vocal characteristics was noted only retrospectively—at the moment of the beep, experientially, Lara simply hears herself talking. She knows who the “you” is in this sentence, but the sentence is not directed to that person. She is also seeing her hands. (Sensory awareness and inner hearing with idiosyncratic characteristics).
(3) (Otto, participant 3, sample 5.5) Otto is saying in inner speech “I just *turned* 30.” He doesn’t know why he is saying that or to whom, but he is clear that that is what he is experiencing and that he is emphasizing the word “turned.” (Inner speaking).
(4) (Susan, participant 4, sample 6.6) Susan innerly sees the actress Sigourney Weaver in a cryogenic tank from the movie *Alien*. She sees Sigourney’s face from above, below the glass window of the tank—the rest of Sigourney’s body is vaguely or blurrily present. Mostly Susan is searching for the word used in the movie: cryogenic chamber, cryogenic tank, etc., waiting for the right word to appear. This is primarily a state of suspended animation, waiting for the word—she does not see or hear pieces of words, etc. (Inner seeing and an uncategorized experience of cognition).
(5) (Jane, participant 5, sample 5.8) Jane is aware of the fidgetiness of her upper trunk, arms, and eyes; they want to move. She is also simultaneously looking at the gray plastic piece of the scanner, noticing especially the dark grayness of it. (Two separate, simultaneous sensory awarenesses).

It can be seen from these examples (and from the entire data set) that resting state experience is active and involved. The resting state is *not* a state of phenomenological rest or suspension, but is typically engaged and explicit. It is sometimes simple but is often complex and multilayered. Sensory awareness can involve any of the senses, can be actual or imaginary, and can be tied to the environment or distant from it. Inner speaking can be self-directed, other directed, or neither; can be in one’s own voice or someone else’s; can be meaningful or relatively meaningless. Similar characterizations of the diversity of experience can be made within inner seeing and feelings.

### Comparing Questionnaire and DES Sampling

We have seen that there were large individual differences in DES-sampled inner experience but small differences in inner experience across the resting state sessions and within sessions. We now inquire about the adequacy of retrospective accounts of resting state experience. Recall that this study began with a typical 5-min resting state study, immediately followed by the participant’s filling out the ReSQ (Delamillieure et al., 2010), a questionnaire designed to characterize inner experiences in the scanner. We now compare the ReSQ (retrospective questionnaire) results with the 5FP (as sampled by DES) results.

The ReSQ is not aimed directly at the 5FP, but two of the ReSQ items seem directly comparable and one is indirectly comparable. The ReSQ presents visual-analog scales which ask participants to rate the resting-state frequency of “visual mental imagery,” which can be taken to be comparable to 5FP inner seeing, and “inner language,” which can be taken to be comparable to 5FP inner speaking. Additionally, the ReSQ asks participants to rate “somatosensory awareness,” which can be taken to be comparable to a subset of the 5FP sensory awareness. Sensory awareness can be visual, auditory, and so on, as well as bodily, so we split the 5FP sensory awareness into bodily sensory awareness and other sensory awareness, and compared the ReSQ somatosensory awareness to bodily sensory awareness. Then we could compare the three ReSQ characterizations to the sampling frequencies. The ReSQ does not inquire about anything related to the two remaining 5FP (unsymbolized thinking or feelings).

**Table 6** presents the ReSQ percentages side by side with the DES (sampling) percentages for the three comparable characteristics for each participant. For example, participant #1 used the visual analog scales of the ReSQ to report that he had spent 40% of his 5-min resting-state time engaged in inner language. His DES sampling showed that 17% of his 36 DES in-scanner samples involved inner speaking.

**Table 6:** Resting-State Questionnaire (first administration) percentages compared to DES sampling percentages.

Participant	Inner speaking		Inner seeing		Bodily sensory awareness	
			
	ReSQ	DES	ReSQ	DES	ReSQ	DES
1	40	17	14	19	38.5	39
2	40.5	22	57.5	22	31.5	36
3	69	53	11	25	78.5	33
4	20	39	60	42	20	8
5	95	14	85	67	42	11
			
Max discrepancy	81.0		35.5		45.5	

The “Max discrepancy” row of **Table 6** shows the largest difference between a participant’s ReSQ percentage and DES percentage. It can be seen that some of these discrepancies are very large; for example, the maximum discrepancy for inner speaking (Participant 5’s) was 81% (her estimate on the ReSQ was 95% minus her 14% obtained by DES).

After participating in 13 DES sampling periods and their 13 expositional interviews, each participant again underwent (in the *Final/post-DES* phase) a standard 5-min resting state scan, immediately followed by another administration of the ReSQ. The second ReSQ maximum discrepancies were substantially smaller than the original ReSQ maximum discrepancies (29.5, 20.5, and 23.5, respectively).

### High Fidelity in a Single Case: Jane’s Inner Experience

We have established that, at least for this small sample, people’s retrospective or general characterizations of their resting state experience are not to be accepted as faithful accounts of actual experience while in the scanner. We have not, however, described that actual experience. We now provide a glimpse into the experience of one participant, described in as high fidelity as we can muster.

We choose to describe participant #5, Jane, because she exemplifies those (perhaps a majority) who are substantially mistaken about their own inner experience. Jane believed, prior to participation, that she talked to herself nearly all the time (as reflected by her self report, by a 90% inner speech rating on a questionnaire not reported here, and by her ReSQ inner speech rating of 95%); however, sampling revealed that she talks to herself only rarely. In the natural environment she had only one example of inner speaking:

(Jane sample 3.4) Jane was in the U-Bahn, sitting on a bench waiting for a train. She is hearing the sound of kids talking—lots of them; she hears their chatter—loud and clear—how they sound, not what they are saying. She is also writing “I cannot see” and saying those words in inner speech, but more attending to the writing.

The inner speaking here is only the third most prominent feature of her experience (after hearing the chatter and attending to the writing).

In the scanner, there was one straightforward example of Jane’s inner speaking:

(Jane sample 5.5) Jane is innerly asking her friend Sharon where she lives in Berlin. Jane is asking this in Swedish (which is both her native tongue and Sharon’s) at a pace that she described as similar to ordinary speaking in that language. Simultaneously Jane, whose eyes are closed, is seeing ambiguous dark shapes in black, yellow, and gold. The two experiences (speaking and seeing colors) are approximately equal in significance/attention.

We counted two samples as containing inner speaking where the speech might not be considered “I thought in words” by most people:

(Jane sample 8.2) Jane is looking at the writing tablet in the mirror and mainly attending to its dark, rectangular shape, which takes up most of the mirror. She can see the piece of paper on it, which is small. The white paper stands out against the dark background. At the moment of the beep she is also innerly saying “Oh yeah!” and thinking that she should email the kindergarten that she had been intending to volunteer at. The thought about the kindergarten is not worded or accompanied by any pictures.

“Oh yeah” was clearly innerly spoken, so we counted it as inner speaking, but the thought was about kindergarten volunteering, and “Oh yeah” was more an accompanying ejaculation (possibly expressing a motivational function of inner speech; McCarthy-Jones and Fernyhough, 2011).

(Jane sample 7.6) Two things are ongoing simultaneously: (1) Jane is seeing the half-moon shape at the top of the scanner mirror. She sees everything that is out there – the people, the tops of computers, etc., particularly noticing the blue/black striped shirt of the person who is walking back and forth. Everything is clear; the striped shirt is more central and more detailed. (2) Jane is imagining herself reading a story aloud to kids. She is growling like a wolf or a bear, and she feels her eyebrows rising for effect, and she feels her fingers straightening out and expanding for terrific effect (in reality, there is no bodily movement). There is a sense of the kids present, but not particular kids (probably kids that she knows).

The growling was oral, so we (liberally) counted it as inner speaking.

There was one sample where inner speaking may have been ongoing:

(Jane sample 6.6) Jane is thinking that her hair was a mess and is simultaneously innerly seeing herself in the mirror in her bathroom at home, a remembering of what she had seen earlier. The thinking portion: she is thinking that her hair is a mess (even though she doesn’t see her hair very completely in the mirror); the thinking involves some hints of words that might be like “Oh man my hair is a mess.” There were not fully formed words, although neither were words completely absent. The seeing portion: the seeing of herself in the mirror includes the seeing of the bathroom, seen through her own eyes, including the mirror, the bright light, the sink, the toilet, and part of her body. There is no specific focus to the image. The thought (of hair being a mess) was the main focus, about 60/40.

There were two samples which involved Jane’s voice being innerly heard (rather than spoken) as an aspect of a recollection. Here is one:

(Jane sample 5.6) Jane is imaginarily replaying a conversation she had with her friend Sharon. Jane innerly sees the floor, sees Sharon in her inner peripheral vision, and hears herself say “If you look at me I don’t look Swedish.” Mostly Jane is paying attention to her own words, which she hears (note that in the original conversation she had spoken the words, but now she hears them, not speaks them).

There were two samples where words were present but which had no experienced meaning. Here’s one:

(Jane sample 7.1) Jane innerly hears herself saying “thinks about what used to be there,” said in her own voice, as clear as hearing someone else externally speaking. She can’t control the speech and it doesn’t present itself as meaningful speech (that is, she doesn’t know *who* thinks, where *there* is, or what *used to be* there). As an equal part of her experience (50:50) Jane simultaneously innerly sees a long piece of something pale and white; simultaneously she somehow knows it to be a log, even though it doesn’t look like a log. That is, she does not see a log. The words she can hear are understood to be related to the image, but she doesn’t know why/how.

If one counts all those samples which contain the experience of words or hints of words—frank inner speech, inchoate inner speech, inner hearing, and so on—the frequency of Jane’s words is 14% in the natural environment and 25% in the scanner, far smaller than her prior-to-sampling self-understanding (90 or 95%). Part of the discrepancy might be accounted for by the fact that Jane frequently engaged in unsymbolized thinking (Hurlburt and Akhter (2008a)): the direct, unambiguous experience of thinking that is not accompanied by words, images, or other symbols (25% in the natural environment and 39% in the scanner). Here, are two examples from the scanner:

(Jane sample 5.1) Jane is wondering whether it is possible to 100% do two things at once (a reference to something RTH had said a few days before). There are no words or symbols, even though she is quite specific about the “100%” part and the “at once” part, etc. She is simultaneously also slightly attending to the scanner sound.

(Jane sample 8.7) Jane’s eyes have closed in a blink and she sees a negative image of the half-moon scanner scene: what is actually white she sees dark, and what is actually dark she sees light pale yellow. This seeing starts where the actual scene exists and then floats downward. Simultaneously she is thinking a little bit about Master’s programs: how courses are selected, how do you decide what thesis to write, how do you write it, etc. This is all without words or images and are all aspects of one thinking.

Hurlburt and Akhter (2008a) reported that many people who have frequent unsymbolized thinking initially believe that such thinking is impossible and (mistakenly) understand themselves to be thinking in words.

Furthermore, Jane’s most frequent kind of experience was sensory awareness (75% in the natural environment and 78% in the scanner). We have given several examples above (samples 3.4, 5.5, 5.8, 7.1, 7.6, and 8.2). Here is another (which includes three simultaneous sensory awarenesses):

(Jane sample 8.8) Jane is seeing the knuckles on her left hand, especially the silvery blue shades of her knuckles caused by the blue light. Simultaneously she hears the incredibly loud noise of the scanner, primarily in her right ear, and she feels her upper body vibrate in sync with that noise.

Hurlburt et al. (2009) reported that many people who have frequent sensory awareness initially (mistakenly) believe that such experience is explicitly cognitive, and (mistakenly) understand themselves to be thinking in words. For example, it is likely that, before sampling, had Jane had the experience described in sample 8.8, she would have (mistakenly) believed that she was saying to herself that the silvery blue shade of her knuckles was interesting.

Over the course of sampling, Jane came to the realization that she frequently had sensory experiences without cognitive overlay and that she frequently had thoughts that did not involve words (what we call unsymbolized thinking). Perhaps as a result, when she characterized her frequency of verbal thinking on the second (end of sampling) administration of the ReSQ, she estimated her resting-state inner speech percentage to be 22.5%, much lower than her first ReSQ estimate of 95%.

## Discussion

We have noted the importance of exploring the experiential phenomena of the resting state, an undertaking that has not been accomplished heretofore because of methodological challenges. We address those challenges using DES, a technique designed to explore experience in high fidelity, in an extended duration resting state fMRI study that involved multiple (nine) sessions, each of long duration (25 min), for a total of 225 min in the scanner rather than the more usual 5 or 10 min.

Because of the intensity of the effort (for each participant, 11 scanner sessions and 13 DES expositional interviews, each with two or more highly skilled interviewers), we used in this exploratory study only five participants. This study is therefore best considered a small first step in an important direction rather than a definitive investigation. All the results that we have reported above and will discuss below must be understood in the context of weighing the risks of small-*n* descriptive studies against their benefits (Hurlburt, 2011a, Chap. 21). The results suggest that the neurophysiological sophistication of the scanner can be profitably combined with the phenomenological sophistication of DES, and that opens substantial possibilities for issues that are central to neuroscience and psychology in general.

Our results indicate that there were not large or systematic shifts in experience across the multiple (nine) sessions or within the long-duration (25 min) sessions. Furthermore, our brain activation results showed that the ALFF patterns in each participant in each of the three phases (the initial 5-min resting state session, the concatenated extended-duration resting state sessions, and the final 5-min resting state session) all showed activation in superior and inferior anterior medial frontal regions, lower precuneus and posterior lateral parietal cortex as would be expected from typical resting state studies. Taken together, these results suggest that our extended-duration resting state sessions were experientially and neurophysiologically broadly similar to typical resting-state sessions.

We found substantial individual differences in resting-state experience across our participants. For example, the resting-state frequency of sensory awareness ranged from 19 to 78%; inner seeing ranged from 19 to 67%; inner speaking ranged from 14 to 53%; and so on. We found similar wide ranges in the natural environment, as did Heavey and Hurlburt (2008). It therefore seems that our five participants were, in broad strokes, similar to what might be expected in a sample of volunteers for psychological or neuropsychological studies. We note that the import of this finding is not diminished by the small sample size: we have documented substantial experiential differences, calling into question the frequently held assumption of universal experiential characteristics.

With the exception of inner seeing, we found a substantial relationship between a person’s experiential frequencies in the natural environment and that person’s in-scanner resting state. At least as measured by the 5FP, our participants apparently engaged in approximately the same forms of experience in the scanner as they do in their own everyday environments—those who have frequent sensory awareness in the natural environment also have frequent sensory awareness in the scanner, and so on. This finding suggests that the term “resting state” may have two unfortunate connotations: that people are psychologically at rest and that there is one state in which they find themselves. Our data suggest that the default mode network (DMN) may be activated because people are *engaging in their usual kinds of spontaneous, everyday experience* in the scanner, the same kind of experience they would engage in if they were actively participating in their own wide-ranging everyday undertakings. That is, the DMN may be active when people experientially do what they usually do (whether resting or not), and is suppressed when the person is instructed by an experimenter to do something foreign or unnatural to that individual. Perhaps scientists characterizing their participants when not engaged in tasks should refer to “unconstrained activity” rather than to the “resting state.”

We emphasize the desirability of replication by others with larger sample sizes. The present study is unique in that it gathers data both in the natural environment and in the scanner, thus affording the first opportunity to compare natural-environment to in-scanner experience. We note the important caveat that the natural environment data gathered here are experiential, so it requires an extrapolation to infer that brain activation, like experience, is similar in the natural environment and the scanner.

As we saw in **Table 1**, researchers have provided a wide variety of characterizations about phenomena in the resting state. Some have noted the verbal nature of this experience (see **Table 1** rows 1 and 2). The present study provided 5 × 36 = 180 glimpses of experience in the resting state; of those, approximately 58 (32%) involved words of any kind, whether innerly spoken, innerly heard or imagined in any other way. The determination of whether a particular sample involves words is not perfectly reliable, so some might say our 32% over-represents or under-represents the frequency of words to some degree, but it is safe to conclude that most of our participant’s sampled resting-state experiences were *not* verbal.

Some researchers have characterized resting state experience as involving planning (**Table 1** rows 3 and 4). Of the present study’s 180 glimpses, approximately 39 (22%) could be said to involve planning when “planning” was defined in a very inclusive way. Here again, the determination of whether planning was part of a particular sample is far from perfectly reliable, but it is safe to conclude that most of our sampled experiences did *not* involve planning for the future.

Some researchers have characterized resting state experience as involving enhanced attention (**Table 1** rows 5 and 6). Our participants did indeed have frequent specific awareness of inner or external stimuli. However, their sensory awarenesses were not *enhanced* or *increased*: our participants had very frequent specific and engrossing sensory awareness in their own natural environments—in fact, the frequency of such experience was slightly *higher* in the natural environment (65.6%) than in the scanner (58.8%). Such naturally occurring sensory awarenesses are very frequently overlooked by people who engage in such phenomena frequently (Hurlburt et al., 2009).

Some researchers have characterized resting state experience as involving an internal monitoring (**Table 1** rows 7, 8, and 9). However, others have held that it involves an external monitoring (**Table 1** row 5), whereas yet others have held that it is an alternation of internal with external (**Table 1** row 6). Our results suggest that actual experience is sometimes inwardly directed, sometimes externally directed, and sometimes neither, with no direction overwhelmingly predominant.

Some researchers have characterized resting state experience in ways that we find difficult to parse in experiential terms (**Table 1** rows 10 and 11). We invite them to peruse our glimpses (all available from us) and draw appropriate conclusions.

We now ask whether the questionnaires designed to investigate resting-state phenomena can do so in high fidelity. The ReSQ asks participants to characterize their experience into five categories (visual imagery, in inner language, in somatosensory awareness, in inner musical experience, and in the mental manipulation of numbers) using instructions that require “that the total score for the five types of activity had to equal 100%” (Delamillieure et al., 2010, p. 566). That instruction presupposes that categories are mutually exclusive: that an experience is either, for example, inner language or somatosensory awareness but not both. Our own participants provide 24 examples (13%) where that mutual-exclusive assumption is far from correct—our Lara (participant 2, sample 7.7) above is one such example (as is example 5, in one respect). Here is another: Otto (sample 7.5) is reciting a poem to himself (Goethe’s “Erlkönig”) and he is currently on the third line (of four) of the seventh stanza (of eight). He is innerly speaking the line “Mein Vater, mein Vater, jetzt faster mich an” in a soft declarative voice. Simultaneously he is counting the stanza on his left fingers and the line within stanza on his right finger (so he is holding his second finger left hand against the desk to indicate seventh stanza and the third finger right hand against the desk to indicate third line). He is more aware of his right hand (apparently because it is about to advance to the fourth line). Simultaneously he is actively trying not to hear the noise of the scanner. That is, he is *not* merely automatically screening out the noise (which he does successfully on other occasions). He hears the noise but he is actively trying not to attend to it. We conclude that the ReSQ presupposition that characteristics must add to 100% is importantly misguided.

The two other questionnaires aimed at characterizing the resting state (ARSQ, Diaz et al., 2013, and ARSQ 2.0, Diaz et al., 2014; and the NYC-Q; Gorgolewski et al., 2014) also have presuppositions that we believe substantially interfere with their ability to ascertain with fidelity the characteristics of experience. For example, both inquire about thoughts. The ARSQ 2.0 instructions ask participants in an online study to sit quietly in front of their computer screen for 5 min. At the conclusion of the 5 min, a screen appears which reads, “The 5 min of rest are over. Now several statements will follow regarding potential feelings and thoughts you may have experienced during the resting period. Please indicate the extent to which you agree with each statement” (Diaz et al., 2014, p. 2). Statements such as “I thought in words” and “I thought about myself” are to be rated on a “five-point ordinal scale with the labels ‘Completely Disagree,’ ‘Disagree,’ ‘Neither Agree nor Disagree,’ ‘Agree,’ and ‘Completely Agree”’ (Diaz et al., 2013, pp. 2–3). The NYC-Q asks participants to report on the content and form of their self-generated thoughts using Likert scales for items that begin “I thought about” *X* or “I thought of” *X* (Gorgolewski et al., 2014, p. 3). However, careful DES interviewing reveals consistently that people use the word “thinking” (or “thought”) in highly disparate ways, leading Hurlburt and Heavey to conclude:

We take it as an axiom that when a DES participant says “I was thinking …,” we know nothing whatever about the phenomena of her inner experience. That claim may be highly counterintuitive, because most people, when asked, do in fact define “thinking” as a cognitive event and correctly discriminate between, for example, “thinking” and “feeling” when observing one person solving a math problem and another crying. Our claim is that when people speak of the experience of others, the referent of thinking is some cognitive process or event, but when they speak of themselves, the referent of thinking is frequently not cognitive and is unspecified and/or unspecifiable.

The word thinking is arguably the most problematic word in the exploration of pristine experience (Hurlburt and Heavey, 2015, p. 151).

Despite the phenomenological ambiguity of the words “thinking” and “thought,” most of the ARSQ items (18 out of 27) and all of the NYC-Q items inquire about thinking as if participants would have a joint understanding about what is being asked. Hurlburt (2011a) held that it requires iterative training to help participants use experiential terminology in consistent ways. For example, suppose that our Jane had had an experience such as her sample 5.8 (fidgetiness noticing the dark grayness of the plastic piece) but had not undergone the DES iterative training. Hurlburt (2011a) would suggest that Jane would very likely have reported herself to have been *thinking* that she wanted to move and *thinking* about the gray plastic piece, rather than to have been engaged in sensory awarenesses of those aspects of her environment.

It might be observed that the DES expositional interviews are themselves retrospective, just like questionnaire reports: both occurred following the participant’s exiting the scanner. However, the DES expositional interviews are constrained by the notes jotted down contemporaneously. One might then observe that even those “contemporaneous” notes are actually retrospective—the target experience was a few seconds prior to the note jotting. This criticism holds that retrospection, no matter how short, so disrupts experience that what *seems* like recollection is actually a construction that is unrelated to whatever experience may have been ongoing at the moment of the beep. We think that is unlikely given our careful questioning of hundreds of people in sampling situations, but we accept that it is possible. However, if that possibility is taken seriously, then *all* first-person reports should be excluded from science, because *all* first-person reports require some sort of retrospection. Excluding all first-person reports is an entirely defensible (although we think misguided) scientific strategy. This issue has been discussed at length in Hurlburt and Schwitzgebel (2007) and Hurlburt (2011a).

It might be observed that the natural environment training performed in this study was more inefficient—even accepting that iterative training is necessary—than performing that iterative training in the scanner, where experiences of exactly the sort that are found in the scanner could be examined and clarified. Hurlburt (2011a) holds that training in a target environment is a risky practice. One of the main objects of the iterative procedure is to help participants overcome or bracket their presuppositions about experience. That is always a difficult thing to accomplish, but it is made more difficult the more closely tied the training is to the target experiences. Furthermore, it would undermine the confidence one might have in the results. For example, suppose that in the present study there had been no natural environment training. Further suppose (as actually happened) that the study discovered exceptionally high frequencies of sensory awareness. In that case, the criticism could have been effectively leveled that the high frequency of sensory awareness was an artifact of the in-scanner training: the noise of the scanner would have been a salient characteristic of the first scanner session, and therefore a large topic of conversation in the first expositional interview. The participant could have gleaned that the interviewer was particularly interested in sensory awareness, and would therefore have been more likely to report sensory awareness in subsequent interviews. However, the study as designed diminishes that criticism. Participants were introduced to sampling in their natural environments, which were no more nor less sensorially salient than usual. The first expositional interviews were not skewed by the experimental situation toward the sensory, and in fact covered a wide range of topics and characteristics absolutely unrelated to scanning features. On the basis of those interviews, we discovered that our participants had sensory interests, but we also conveyed by word and deed to our participants that we were not *particularly* interested in sensory aspects. With that as background, we think that whatever phenomena are discovered in the in-scanner sampling are more believable.

It might be observed, following Ross and Nisbett (1991), that the intensive, multiple interviews such as those we have conducted run the risk of biasing the participant. That is indeed a risk, but Hurlburt has written extensively (Hurlburt and Heavey, 2006; Hurlburt, 2011a) about the many ways DES manages that risk. For one example, the DES procedure is “open-beginninged” (Hurlburt, 2011a): the interviewer does not initially inquire about any phenomenon that has been specified *a priori* (that is, DES does *not* initially inquire about images, or about inner speech, or about any other pre-defined topic) but instead asks for a description of whatever experience, if any, was ongoing at the moment of the beep, and then follows up on the participant’s response. Hurlburt (2007, pp. 285–289) has argued that Ross and Nisbett’s own analysis shows that “opening up the channel factor” mitigates the obedience risk, and Hurlburt has shown how DES gives multiple and repeated channel-opening instructions:

For example, we explicitly and repeatedly told (DES participant) Melanie that she could withdraw at any time; that saying “I don’t know” or “I don’t remember” was a perfectly legitimate response, that we valued her best effort over any predetermined expectation; that it was quite possible that things wouldn’t be clear and that that was okay; that the task was perhaps impossible; that we would learn as much or more from her inability to perform a task as we would from her ability to perform it easily; that we much preferred her unexaggerated candor to any attempt to figure out what we wanted to hear; and so on. Not only did we say such things repeatedly, but we meant them sincerely; and not only did we mean them sincerely, I think Melanie recognized that we meant them sincerely. Therefore, by Ross and Nisbett’s own argument, we, I think, successfully undermined the channel effect and therefore should *not* expect large obedience effects.

(Hurlburt, 2007, p. 288)

It might be asked whether the intensive training provided in this study alters the participants so that their resting states are no longer similar to untrained individuals. We do indeed believe that participants become more skilled at apprehending their experience, but for most participants, we think that does not substantially alter the nature of their experience. **Table 4** provides a bit of support for that statement: there is not substantial experiential drift across the sessions. Replication by multiple methods is desirable.

Even if one accepts the fidelity of the individual observations, it might be observed that because of the small sample size (*n* = 5), generalization to any population is risky. That is indeed true (and we have tried to insert appropriate caveats to that effect throughout), but there are advantages and disadvantages to all approaches in science including large sample size. We take as an example the Diaz et al. (2014) briefly described above, because it is an exemplary study at the state of its art and one which appeared recently in this journal.

Diaz et al. (2014) had a large sample (1444 participants) fill out the ARSQ or ARSQ 2.0 to investigate “mind-wandering experiences,” including “verbal thought.” The disadvantage of large *n* is that Diaz et al. (2014) cannot know what any participant intended when endorsing *Agree* to any of the ARSQ statements, for example, to “I thought in words.” Participants might endorse *Agree* because they thought in words *frequently*, or because they thought in words *once* but recall it, or because they mistakenly believe themselves to think in words even though they *never* actually thought in words during the experiment. To sort through those important differences requires an iterative method (Hurlburt, 2011a), which in turn requires multiple interviews and multiple skilled interviewers, making large sample sizes impossible.

Questionnaire responses can be validated in a variety of ways (see Alderson-Day and Fernyhough, 2015, for a review of inner speech validation), and such validation is necessary to science. However, Hurlburt (2011a, Chap. 21) argued in favor of a science that values both validity-based and observation-based methods and that recognizes the very different constraints that operate in each realm. Here, for example, we discovered that one of our five participants, Jane, firmly believed herself (prior to participation) to be a nearly-all-the-time inner speaker but was more likely a nearly-none-of-the-time inner speaker. Does that imply that 20% of people are hugely mistaken about their inner speech? Certainly not—such a claim would indeed require a large *n* study. However, it does show that *some* people (Hurlburt, 2011a, believes that Jane is by no means exceptional) are hugely mistaken. Furthermore, we have shown that a few samples from one person can provide rich insight into the nature of verbal experience—that words are sometimes spoken, sometimes heard, sometimes inchoate, sometimes meaningless. That is a revealed phenomenological richness that cannot possibly be obtained from questionnaires, even from thousands of responses to “Please indicate the extent to which you agree with ‘I thought in words’.” Such results can be obtained only from idiographic, iterative examination of individuals (Hurlburt, 2011a), and such depth of examination and careful disentangling of language is possible only with small numbers of participants.

This was a small study in terms of number of participants, but a large study in terms of intensity [13 DES expositional interviews, 11 scanner sessions (approximately 275 min) per participant]. The results are provocative: they suggest that high fidelity descriptions of inner experience can be gathered in the scanner; they suggest that there are large individual differences in inner experience in the scanner; they suggest that experience in the resting state may be characterized as being unconstrained activity. Clearly these results need to be replicated by larger studies and by investigators unrelated to us, but if replicated, they would make significant contributions to scientists’ quest to integrate information about experience and brain function in the MRI scanner.

## Conflict of Interest Statement

The authors declare that the research was conducted in the absence of any commercial or financial relationships that could be construed as a potential conflict of interest.

## Footnotes

1 www.adni-info.org

2 https://www.nitrc.org/projects/mricron
